# Survey of Emergency Department Clinicians on the Utility of the Guaiac Fecal Occult Blood Test

**DOI:** 10.7759/cureus.70922

**Published:** 2024-10-06

**Authors:** Ehsan Shirazi, Albertha v Lalljie, Michael G Heckman, Alexander Hochwald, Osman Hamid, Elmkdad Mohammed, Johnathan M Sheele

**Affiliations:** 1 Emergency Medicine, Mayo Clinic, Jacksonville, USA; 2 Biostatistics, Mayo Clinic, Jacksonville, USA; 3 Clinical Trials and Biostatistics, Mayo Clinic, Jacksonville, USA; 4 St. Elizabeth Family Medicine Residency Program, Mohawk Valley Health System - St. Elizabeth Campus, Utica, USA

**Keywords:** acute gastrointestinal tract bleeding, guaiac fecal occult blood test, hematochezia, melena, undifferentiated anemia

## Abstract

Introduction: Emergency department (ED) clinicians utilize the guaiac fecal occult blood test (gFOBT) in their assessment of suspected gastrointestinal bleeding or unexplained anemia despite supporting evidence. ED clinicians' ability to predict the gFOBT results and how the gFOBT results could affect ED patient disposition has not been previously studied.

Methods: From October 16, 2019, through September 15, 2020, we conducted a single-site survey of ED clinicians before and after performing gFOBTs during routine clinical care. Survey data were collected and retrospectively evaluated with unadjusted and multivariable regression analyses.

Results: We examined a total of 133 combined pre-gFOBT and post-gFOBT surveys. ED clinicians accurately predicted gFOBT results with an area under the receiver operating characteristic curve of 0.75 (95%CI, 0.66-0.85). Of clinician-predicted certain or very probable positive gFOBT results, only 79% were actually positive. In multivariable analyses, decreased hematocrit level (odds ratio (OR), 0.31/10% increase; 95%CI, 0.14-0.61), decreased red blood cell count (OR, 0.41/1x10^12^/L increase; 95%CI, 0.21-0.75), and absence of firm stool consistency (OR, 0.09; 95%CI, 0.01-0.42) were associated with positive gFOBT results (all P<.006). The most common reason for performing gFOBTs was black stool or suspected melena, followed by decreased hemoglobin level, red blood in stool, and suspected upper gastrointestinal tract bleeding. Before performing gFOBT, 50.8% of clinicians responded that the test results would change patient disposition, which decreased to 30.5% after the gFOBT result.

Conclusions: We found that ED clinicians cannot predict the gFOBT results with high accuracy. A suspected GI bleed is the main reason for performing the test in the ED.

## Introduction

Potential gastrointestinal (GI) tract bleeding episodes account for approximately 0.3% of all emergency department (ED) visits in the United States [1]. ED providers may utilize a fecal occult blood test (FOBT) to check for GI bleeding despite evidence supporting this practice. The FOBT has been used for decades to screen for GI tract bleeding as a marker of colorectal cancer [2]. Currently, two primary types of FOBTs are in use: the fecal immunochemical test, which is not readily available in EDs, and the guaiac FOBT (gFOBT). The gFOBT detects the pseudoperoxidase activity of the heme component of hemoglobin in stool. However, plant peroxidases, food dyes, and certain medications may lead to false-positive and false-negative gFOBT results [3-5]. Some dietary or drug restrictions may be required to optimize the accuracy of the gFOBT results. In addition, some food dyes and medications can change the color of stool, further complicating the assessment of GI tract bleeding in the ED [5].

Although the gFOBT is a validated screening tool for colorectal cancer, it is also used in the ED and for inpatients to assess for acute GI tract bleeding or undifferentiated anemia. The off-label use of gFOBT in the ED needs more research. Investigations of gFOBT in the primary care setting and for hospitalized patients have concluded that it is of limited value outside of cancer screening [6-9]. Some investigators have called for the removal of gFOBT from hospitals and EDs [8,10].

Obtaining stool for the gFOBT specimen by digital rectal examination (DRE) is uncomfortable for patients and clinicians. If the gFOBT has limited diagnostic utility in the ED, the test potentially could be avoided. The objective of our study was to compare ED clinician-predicted gFOBT results with the actual gFOBT results. We also examined potential confounding factors of ED patient disposition for patients undergoing the gFOBT and the variables associated with positive gFOBT results.

## Materials and methods

Participant selection and study design

This study was performed according to Strengthening the Reporting of Observational Studies in Epidemiology (STROBE) guidelines [11]. We prospectively surveyed clinicians before and after they performed the gFOBT (Hemoccult Sensa, Beckman Coulter, Inc., Brea, California, United States) in the ED at Mayo Clinic in Jacksonville, Florida, 24 hours a day, seven days a week, from October 16, 2019, through September 15, 2020. The Mayo Clinic Institutional Review Board approved this study (approval number: 10429). Participation in the study was voluntary and informed written consent was obtained from all ED clinicians before completing each survey. The requirement for informed consent was waived for patients.

All ED clinicians, which included advanced practice providers and attendings performing a DRE and gFOBT as part of their clinical workup during the study period, were asked to self-complete the surveys (See Appendices). ED clinicians were reminded about voluntary participation in the study through regular departmental emails and signs posted in the ED. The paper surveys were kept near the gFOBT testing materials, and completed surveys were stored in a locked box and collected weekly. The pre-gFOBT and post-gFOBT surveys were submitted as a single completed survey. Survey data was included only of ED patients ≥18 years of age.

Survey results were transcribed and managed using the REDCap electronic data capture tools hosted at Mayo Clinic [12,13]. We also retrospectively searched our electronic health records for patient information that corresponded to each completed gFOBT survey. This patient information included demographics, triage status, laboratory findings, and diagnoses.

Statistical analysis

Continuous variables were summarized as median (range), and categorical variables were summarized as frequency (percentage). A Wilcoxon rank sum test was used to compare the ED clinician-predicted likelihood of positive gFOBT results between actual positive (weakly, moderately, or strongly positive) or negative gFOBT result groups. Unadjusted and multivariable logistic regression models were used to evaluate associations of patient and GI tract characteristics with the separate outcomes of whether the result of the gFOBT changed the plan for disposition and the occurrence of a positive test result (only in patients with sufficient stool reported). Multivariable models were adjusted for variables associated with the given outcome with a P value <.05 in unadjusted analyses. For the occurrence of a positive test result, all variables with a P value <.05 in the unadjusted analyses were not adjusted for due to the rare nature of this outcome. According to the recommended guidelines, we adjusted only for variables with the strongest associations in the unadjusted analyses [14]. No adjustments for multiple testing were made in these exploratory analyses, and P values <.05 were considered statistically significant. All statistical tests were two-sided. Statistical analysis was performed with R statistical software, v4.0.3 (R Foundation for Statistical Computing, Vienna, Austria).

## Results

We analyzed 133 of 136 completed surveys; three were excluded because of missing patient identifying information. Two patients in our cohort underwent two separate DREs with a gFOBT during the study period, and data from both ED visits for each patient were included in our analyses. Patient characteristics are listed in Table 1. The median (range) age was 71 (19-101) years. Patient dispositions were approximately evenly distributed; 39.5% of patients were admitted to the hospital, 24.2% underwent hospital observation, and 36.3% were discharged.

**Table 1:** Patient characteristics (N=133 Surveys) BP, blood pressure; ED, emergency department; MCH, mean corpuscular hemoglobin; MCHC, mean corpuscular hemoglobin concentration; MCV, mean corpuscular volume; RBC, red blood cell; RDW-CV, red cell distribution width; RDW-SD, red cell distribution width standard deviation; BUN, blood urea nitrogen. Categorical variables (sex, method of arrival, and patient disposition) are summarized as No. (%) of patients, and all other variables are summarized as median (range) ^b^ Shock index calculated as heart rate divided by systolic blood pressure.

Characteristic	Number of surveys	Value, median (range)
Age at visit (years)	126	71 (19-101)
Sex	124	
Men		60 (48.4)
Women		64 (51.6)
Method of arrival to ED	123	
Ambulance		28 (22.8)
Not known to be ambulance		95 (77.2)
Patient disposition	124	
Admission		49 (39.5)
Hospital observation		30 (24.2)
Discharge		45 (36.3)
Systolic BP (mm Hg)	124	144 (87-215)
Diastolic BP (mm Hg)	124	76 (32-119)
Mean arterial pressure (mm Hg)	124	98.3 (50.7-148.3)
Shock index^b^	122	0.6 (0.4-1.4)
Pulse pressure (mm Hg)	124	63 (12-139)
Pulse (beats/minute)	122	89 (60-138)
BUN (mg/dL)	119	21 (5-119)
Hematocrit (%)	122	32.0 (6.8-50.6)
MCHC (g/dL)	121	32.0 (24.7-36.2)
MCH (pg/cell)	121	30.0 (15.1-37.0)
MCV (fL)	121	93.3 (61.1-121.4)
Platelet count (×10^9^/L)	120	230 (23-664)
RBC count (×10^12^/L)	121	3.4 (1.5-6.6)
RDW-CV (%)	121	14.9 (11.9-24.9)
RDW-SD (fL)	121	50.7 (33.7-84.1)

ED clinician responses to surveys conducted before and after the gFOBT are summarized in Table 2. In the pre-gFOBT surveys, clinicians responded that more patients had formed stools (49.1%) than liquid (24.5%) or somewhat formed (26.4%) stools. Most patients (82.6%) had no vomiting. ED clinicians reported that the most common reason for performing the gFOBT was because the patient had black stool or suspected melena, followed by red blood in stool, dropping hemoglobin level, and suspected upper GI tract bleeding.

**Table 2:** Survey results before and after gFOBT (N=133) DRE, digital rectal examination; gFOBT, guaiac fecal occult blood test; GI, gastrointestinal; INR, international normalized ratio ^a^ Multiple reasons for performing DRE/gFOBT could be selected by survey respondents.

Survey response	Number of surveys	Frequency (percentage)
Pre-gFOBT Survey		
Number of stools in the past 24 hours	97	
0		3 (3.1%)
1		34 (35.1%)
2		20 (20.6%)
³3		40 (41.2%)
Last stool consistency	110	
All liquid		27 (24.5%)
Somewhat formed		29 (26.4%)
Formed		54 (49.1%)
Took bismuth subsalicylate in past 48 hours	115	6 (5.2)
Number of bouts of emesis in past 24 hours	121	
0		100 (82.6%)
1		7 (5.8%)
2		2 (1.7%)
3		12 (9.9%)
Coffee ground or bloody emesis	130	15 (11.5%)
First most important reason for performing DRE/gFOBT	133	
Black stool or suspected melena		55 (41.4%)
Decreased hemoglobin level		32 (24.1%)
Red blood in stool		30 (22.6%)
Suspected upper GI tract bleeding		11 (8.3%)
Other		5 (3.8%)
Second most important reason for performing DRE/gFOBT	66	
Black stool or suspected melena		10 (15.2%)
Decreased hemoglobin level		11 (16.7%)
History of GI tract bleeding		16 (24.2%)
Red blood in stool		7 (10.6%)
Suspected upper GI tract bleeding		10 (15.2%)
Other		12 (18.2%)
Third most important reason for performing DRE/gFOBT	25	
Decreased hemoglobin level		5 (20.0%)
History of GI tract bleeding		4 (16.0%)
Suspected upper GI tract bleeding		8 (32.0%)
Other		8 (32.0%)
Reasons for performing DRE/gFOBT^a^	133	
Black stool or suspected melena		65 (48.9%)
Red blood in stool		38 (28.6%)
Suspected upper GI tract bleeding		30 (22.6%)
Decreased hemoglobin level		48 (36.1%)
Examination of hemorrhoid		3 (2.3%)
Examination of rectal mass		1 (0.8%)
Distinguish from genital or genitourinary tract bleeding source		2 (1.5%)
High INR (or anticoagulant use) and possible GI tract bleeding		7 (5.3%)
History of GI tract bleeding		23 (17.3%)
Unstable vital signs		4 (3.0%)
Low platelet count		1 (0.8%)
Unexplained weight loss		0 (0.0%)
Screening for colon cancer		0 (0.0%)
Other		8 (6.0%)
Predicted gFOBT-instigated change in patient disposition	130	66 (50.8%)
Predicted likelihood of positive gFOBT result	133	
No chance		2 (1.5%)
Slight possibility		32 (24.1%)
Fair possibility		29 (21.8%)
Very probable		41 (30.8%)
Certain		29 (21.8%)
Post-gFOBT Survey		
gFOBT result	132	
Negative		54 (40.9%)
Weakly positive		6 (4.5%)
Moderately positive		15 (11.4%)
Strongly positive		57 (43.2%)
Predominant stool color	126	
Brown		55 (43.7%)
Black		37 (29.4%)
Red/pink		18 (14.3%)
Yellow		9 (7.1%)
Orange		3 (2.4%)
Other		4 (3.2%)
Amount of stool obtained for the gFOBT	133	
None		6 (4.5%)
Very little		32 (24.1)
Sufficient		95 (71.4)
Stool consistency	125	
Tarry/thick		26 (20.8)
Liquid		57 (45.6)
Firm		42 (33.6)
Presence of a hemorrhoid	133	
Yes		13 (9.8)
No		119 (89.5)
Unsure		1 (0.8)
Presence of a rectal fissure	132	
Yes		0 (0.0)
No		131 (99.2)
Unsure		1 (0.8)
Reported post-gFOBT results changed patient disposition	131	
Yes		40 (30.5)
No		91 (69.5)

The percentage of ED clinician-predicted positive gFOBT results significantly differed between patients with an actual positive gFOBT result vs those with a negative test result (P<.001) (Table 3). Of 29 gFOBT results with a predicted certain likelihood of positivity, 23 (79%) were actually positive. Of 41 with a very probable prediction, 32 (78%) were positive. Of 29 with a fair possibility of positivity, 16 (55%) were positive. Of 31 with a slight possibility, seven (23%) were positive, and of two predicted to have no chance of positivity, none (0%) were positive. Clinicians predicted positive and negative gFOBT results with an area under the receiver operating characteristic curve of 0.75 (95%CI, 0.66-0.85).

**Table 3:** Comparison of emergency department clinician–predicted gFOBT results with the actual results gFOBT: guaiac fecal occult blood test ^a^P value determined by Wilcoxon rank sum test

Predicted likelihood of positivity	Positive result (n=78), n (%)	Negative result (n=54), n (%)	P^a^
No chance	0 (0.0)	2 (3.7)	< .001
Slight possibility	7 (9.0)	24 (44.4)	
Fair possibility	16 (20.5)	13 (24.1)	
Very probable	32 (41.0)	9 (16.7)	
Certain	23 (29.5)	6 (11.1)	

In both unadjusted and multivariable analyses, the only variables associated with ED clinician survey responses reporting that the gFOBT results would change patient disposition were black stool or suspected melena as the most important reason for performing the DRE (odds ratio (OR), 3.05; 95%CI, 1.43-6.69; P=.004) or as one of the reasons for performing the DRE (OR, 3.17; 95%CI, 1.47-7.12; P=.004) (Table 4). In unadjusted analyses, lower hematocrit level (OR, 0.41/10% increase; 95%CI, 0.21-0.75; P=.006), lower red blood cell count (OR, 0.44/1 ´ 10^12^/L increase; 95%CI, 0.24-0.76; P=.005), absence of firm stool consistency (OR, 0.06; 95%CI, 0.01-0.26; P<.001), and lack of primarily brown stool color (OR, 0.27; 95%CI, 0.11-0.65; P=.004) were associated with a positive gFOBT result (Table 5). In multivariable analysis, decreased mean corpuscular volume (OR, 0.47/10-fL increase; 95%CI, 0.22-0.95; P=.04), lower hematocrit level (OR, 0.31/10% increase; 95%CI, 0.14-0.61; P=.001), lower red blood cell count (OR, 0.41/1-unit increase; 95%CI, 0.21-0.75; P=.006), and lack of a firm stool consistency (OR, 0.09; 95%CI, 0.01-0.42; P=.006) were all associated with a positive gFOBT result.

**Table 4:** Variables associated with whether gFOBT results changed the plan for patient disposition BP, blood pressure; DRE, digital rectal examination; ED, emergency department; GI, gastrointestinal; MCH, mean corpuscular hemoglobin; MCHC, mean corpuscular hemoglobin concentration; MCV, mean corpuscular volume; OR, odds ratio; RBC, red blood cell; RDW-CV, red cell distribution width; RDW-SD, red cell distribution width standard deviation; BUN, blood urea nitrogen; gFOBT, guaiac fecal occult blood test ^a^ Multivariable models were adjusted for variables that were associated with the outcome with P<.05 in the unadjusted analysis. Specifically, all models were adjusted for black stool or suspected melena being a reason for performing DRE. We also did not adjust for black stool or suspected melena being the most important reason for performing DRE because of its high degree of association with the former variable. ^b^ ORs, 95% CIs, and P values derived from logistic regression models. ORs correspond to the increase given in parentheses (continuous and ordinal variables) or to the presence of the given characteristic (categorical variables).

		Median (range)/Number of patients (percentage)		Unadjusted analysis		Multivariable analysis^a^	
Variable	Number of surveys	Result did not change the plan for disposition (n=91)	Result changed the plan for disposition (n=40)	OR (95% CI)^b^	P	OR (95% CI)^b^	P
Age at visit (per 10-year increase)	126	7.0 (1.9-10.1)	7.1 (2.9-9.4)	1.03 (0.83-1.30)	.79	1.06 (0.83-1.36)	.66
Sex (reference: male)	122	40 (46.5)	19 (52.8)	1.29 (0.59-2.82)	.53	1.59 (0.69-3.72)	.28
Means of arrival (reference: ambulance)	121	21 (24.7)	7 (19.4)	1.36 (0.54-3.77)	.53	1.01 (0.37- 2.96)	.98
Systolic BP (per 10-mm Hg increase)	122	14.4 (8.7-21.5)	14.4 (8.8-18.9)	1.02 (0.89-1.18)	.73	1.06 (0.92-1.23)	.42
Diastolic BP (per 10-mm Hg increase)	122	7.3 (4.5-11.9)	8.0 (3.2-11.2)	1.07 (0.84-1.37)	.58	1.15 (0.89-1.50)	.29
Mean arterial BP (per 10-mm Hg increase)	122	9.6 (6.3-14.8)	9.9 (5.1-12.9)	1.06 (0.85-1.32)	.61	1.13 (0.90-1.44)	.29
Shock index (per 0.1-unit increase)	120	6.1 (3.8-13.7)	5.7 (3.6-10.9)	0.96 (0.78-1.16)	.68	0.95 (0.76-1.17)	.64
Pulse pressure (per 10-unit increase)	122	6.2 (1.2-13.9)	6.2 (1.6-12.3)	0.96 (0.70-1.29)	.79	1.02 (0.86-1.22)	.80
Pulse (per 10-beat/min increase)	120	8.8 (6.0-13.0)	8.9 (6.0-13.8)	1.00 (0.85-1.19)	.96	1.03 (0.74-1.42)	.84
BUN (per doubling mg/dL)	117	4.5 (2.3-6.9)	4.2 (2.6-6.4)	0.70 (0.45-1.07)	.10	0.69 (0.43-1.07)	.10
Hematocrit (per 10% increase)	120	3.1 (0.7-5.1)	3.5 (2.1-4.9)	1.46 (0.93-2.35)	.11	1.39 (0.85-2.33)	.20
MCHC (per 1-g/dL increase)	119	31.8 (26.9-36.2)	32.0 (24.7-34.6)	1.05 (0.85-1.33)	.65	1.03 (0.81-1.31)	.84
MCH (per 1-pg/cell increase)	119	29.9 (17.9-36.1)	30.5 (15.1-37.0)	1.04 (0.94-1.17)	.43	1.03 (0.93-1.15)	.61
MCV (per 10-fL increase)	119	9.3 (6.2-11.5)	9.5 (6.1-12.1)	1.06 (0.85-1.32)	.61	1.11 (0.73-1.72)	.63
Platelet count (per 100 ´ 10^9^/L increase)	118	2.3 (0.2-6.6)	2.3 (0.7-5.5)	1.05 (0.73-1.49)	.80	1.08 (0.74-1.57)	.69
RBC count (per 1 ´ 10^12^/L increase)	119	3.3 (1.5-6.6)	3.7 (1.7-5.0)	1.24 (0.84-1.85)	.28	1.20 (0.79-1.84)	.39
RDW-CV (per 5% increase)	119	3.0 (2.4-5.0)	2.9 (2.4-4.6)	0.83 (0.39-1.67)	.61	0.89 (0.39-1.91)	.77
RDW-SD (per 10-fL increase)	119	5.1 (3.4-8.4)	5.0 (4.0-6.7)	0.98 (0.63-1.48)	.93	1.02 (0.63-1.59)	.95
Number of stools in the last 24 hours (per 1-stool increase)	96	2.0 (0.0-3.0)	2.0 (0.0-3.0)	0.98 (0.62-1.56)	.94	0.89 (0.55-1.44)	.64
Last stool consistency	108			Overall test of difference: P=.90		Overall test of difference: P=.90	
All liquid		18 (26.1)	9 (23.1)	1.00 (reference)	NA	1.00 (reference)	NA
Somewhat formed		17 (24.6)	11 (28.2)	1.29 (0.43-3.97)	.65	1.21 (0.39-3.79)	.75
Formed		34 (49.3)	19 (48.7)	1.12 (0.43-3.05)	.82	1.40 (0.51-4.04)	.52
Took bismuth subsalicylate in past 48 hours	114	2 (2.6)	3 (8.1)	3.31 (0.53-26.00)	.20	2.05 (0.31-16.54)	.45
No. bouts of emesis in the last 24 hours (reference: >0)	120	20 (22.0)	12 (30.0)	1.52 (0.65-3.50)	.33	1.45 (0.60-3.44)	.40
Coffee ground or bloody emesis	128	9 (9.9)	5 (12.5)	1.30 (0.38-4.05)	.66	1.43 (0.40-4.70)	.57
Most important reason for performing DRE	131						
Black stool/suspected melena		30 (33.0)	24 (60.0)	3.05 (1.43-6.69)	.004	3.05 (1.43-6.69)	.004
Dropping hemoglobin		26 (28.6)	6 (15.0)	0.44 (0.15-1.12)	.10	0.73 (0.23-2.10)	.57
Red blood in stool		25 (27.5)	5 (12.5)	0.38 (0.12-1.00)	.07	0.59 (0.18-1.75)	.36
Suspected upper GI tract bleed		7 (7.7)	3 (7.5)	0.97 (0.20-3.71)	.97	1.71 (0.33-7.25)	.48
Other		3 (3.3)	2 (5.0)	1.54 (0.20-9.68)	.64	3.15 (0.38-21.3)	.24
One of the reasons for performing DRE^c^	131						
Black stool/suspected melena		36 (39.6)	27 (67.5)	3.17 (1.47-7.12)	.004	3.17 (1.47-7.12)	.004
Red blood in stool		30 (33.0)	8 (20.0)	0.51 (0.20-1.20)	.14	0.69 (0.26-1.74)	.45
Suspected upper GI tract bleed		18 (19.8)	11 (27.5)	1.54 (0.63-3.63)	.33	1.35 (0.54-3.29)	.51
Dropping hemoglobin		38 (41.8)	10 (25.0)	0.46 (0.20-1.04)	.07	0.58 (0.24-1.34)	.21
History of GI tract bleed		16 (17.6)	7 (17.5)	0.99 (0.35-2.57)	.99	0.83 (0.28-2.22)	.71
Predominant stool color (reference: brown)	130	54 (60.0)	22 (55.0)	0.81 (0.38-1.74)	.59	0.71 (0.32-1.56)	.39
Stool consistency				Overall test of difference: P=.75		Overall test of difference: P=.75	
Tarry/thick	124	17 (20.0)	9 (23.1)	1.00 (reference)	NA	1.00 (reference)	NA
Liquid	131	38 (41.8)	16 (40.0)	0.93 (0.43-1.97)	.85	1.22 (0.54-2.76)	.63
Firm	124	63 (69.2)	31 (77.5)	1.53 (0.66-3.80)	.34	1.16 (0.47-2.98)	.75
Hemorrhoid present on DRE	131	7 (7.7)	6 (15.0)	2.12 (0.64-6.83)	.21	2.08 (0.60-7.04)	.24

**Table 5:** Variables associated with positive gFOBT results for patients with sufficient stool for testing BP, blood pressure; DRE, digital rectal examination; ED, emergency department; GI, gastrointestinal; MCH, mean corpuscular hemoglobin; MCHC, mean corpuscular hemoglobin concentration; MCV, mean corpuscular volume; OR, odds ratio; RBC, red blood cell; RDW-CV, red cell distribution width; RDW-SD, red cell distribution width standard deviation; BUN, blood urea nitrogen; gFOBT, guaiac fecal occult blood test ^a^ Multivariable models were adjusted for variables that were associated with the outcome with P<.05 in the unadjusted analysis. Specifically, all models were adjusted for black stool or suspected melena being a reason for performing DRE. We also did not adjust for black stool or suspected melena being the most important reason for performing DRE because of its high degree of association with the former variable. ^b^ ORs, 95% CIs, and P values derived from logistic regression models. ORs correspond to the increase given in parentheses (continuous and ordinal variables) or to the presence of the given characteristic (categorical variables).

		Median (range)/Number of patients (percentage)		Unadjusted analysis		Multivariable analysis^a^	
Variable	Number of surveys	Negative result (n=32)	Positive result (n=62)	OR (95% CI)^b^	P	OR (95% CI)^b^	P
Age at visit (per 10-year increase)	88	7.3 (3.8-9.9)	7.4 (1.9-10.1)	0.98 (0.74-1.29)	.87	0.99 (0.96-1.02)	.57
Sex (reference: male)	87	16 (53.3)	25 (43.9)	0.68 (0.28-1.66)	.40	0.81 (0.29-2.28)	.69
Means of arrival (reference: ambulance)	87	9 (30.0)	11 (19.3)	1.79 (0.64-5.00)	.26	2.06 (0.64-6.79)	.22
Systolic BP (per 10-mm Hg increase)	87	14.6 (9.5-21.5)	14.2 (8.7-20.4)	0.93 (0.80-1.08)	.37	0.96 (0.80-1.14)	.62
Diastolic BP (per 10-mm Hg increase)	87	7.4 (4.5-11.5)	7.2 (3.2-11.7)	0.89 (0.67-1.18)	.43	0.99 (0.72-1.37)	.96
Mean arterial BP (per 10-mm Hg increase)	87	9.3 (6.3-14.8)	9.8 (5.1-13.0)	0.89 (0.69-1.13)	.34	0.96 (0.72-1.27)	.77
Shock index (per 0.1-unit increase)	85	5.6 (3.6-10.2)	6.2 (3.8-12.0)	1.11 (0.89-1.42)	.36	1.10 (0.85-1.45)	.48
Pulse pressure (per 10-unit increase)	87	7.1 (1.6-11.4)	6.2 (2.1-13.9)	0.95 (0.78-1.14)	.56	0.94 (0.75-1.17)	.55
Pulse (per 10-beat/min increase)	85	8.2 (6.0-13.8)	8.9 (6.0-12.7)	1.08 (0.79-1.52)	.62	1.15 (0.80-1.67)	.44
BUN (per doubling mg/dL)	83	4.2 (2.3-6.1)	4.8 (2.6-6.9)	1.52 (0.95-2.51)	.09	1.58 (0.91-2.85)	.11
Hematocrit (per 10% increase)	86	3.8 (1.8-4.9)	3.1 (0.7-5.1)	0.41 (0.21-0.75)	.006	0.31 (0.14-0.61)	.001
MCHC (per 1-g/dL increase)	85	32.2 (29.0-34.6)	32.3 (27.1-36.2)	0.99 (0.76-1.29)	.95	0.86 (0.63-1.15)	.31
MCH (per 1-pg/cell increase)	85	30.5 (23.5-34.9)	30.8 (19.4-36.1)	0.98 (0.85-1.12)	.80	0.84 (0.70-0.99)	.05
MCV (per 10-fL increase)	85	9.5 (8.1-10.8)	9.5 (7.1-11.0)	0.92 (0.52-1.60)	.78	0.47 (0.22-0.95)	.04
Platelet count (per 100 ´ 10^9^/L increase)	84	2.2 (0.2-5.8)	2.2 (0.5-6.6)	0.92 (0.62-1.38)	.68	1.03 (0.65-1.62)	.91
RBC count (per 1 ´ 10^12^/L increase)	85	4.1 (1.8-5.2)	3.2 (1.7-5.6)	0.44 (0.24-0.76)	.005	0.41 (0.21-0.75)	.006
RDW-CV (per 5% increase)	85	2.8 (2.4-4.5)	3.1 (2.4-4.6)	1.50 (0.62-3.92)	.38	1.78 (0.66-5.23)	.27
RDW-SD (per 10-fL increase)	85	4.9 (4.0-8.4)	5.1 (3.9-7.6)	1.12 (0.67-1.90)	.68	0.93 (0.50-1.69)	.82
No. of stools in the last 24 h (per 1-stool increase)	67	2.0 (0.0-3.0)	2.0 (0.0-3.0)	1.35 (0.79-2.35)	.27	1.06 (0.54-2.02)	.86
Last stool consistency	80			Overall test of difference: P=.047		Overall test of difference: P=.03	
All liquid		4 (16.0)	18 (32.7)	1.00 (reference)	NA	1.00 (reference)	NA
Somewhat formed		5 (20.0)	17 (30.9)	0.76 (0.16-3.32)	.71	1.13 (0.19-6.76)	.89
Formed		16 (64.0)	20 (36.4)	0.28 (0.07-0.92)	.048	1.07 (0.18-6.63)	.94
Took bismuth subsalicylate in past 48 h	81	2 (7.4)	3 (5.6)	0.74 (0.11-5.85)	.75	0.64 (0.08-6.08)	.67
No. of bouts of emesis in the past 24 h (reference: >0)	94	11 (34.4)	11 (17.7)	0.41 (0.15-1.10)	.08	0.49 (0.16-1.47)	.20
Coffee ground or bloody emesis	94	6 (18.8)	4 (6.5)	0.30 (0.07-1.13)	.08	0.38 (0.07-1.80)	.23
Most important reason for performing DRE	94						
Black stool/suspected melena		17 (53.1)	29 (46.8)	0.78 (0.33-1.82)	.56	0.21 (0.06-0.66)	.01
Dropping hemoglobin		7 (21.9)	13 (21.0)	0.95 (0.34-2.80)	.92	2.87 (0.86-10.86)	.10
Red blood in stool		3 (9.4)	15 (24.2)	3.09 (0.92-14.15)	.10	2.69 (0.68-13.52)	.18
Suspected upper GI tract bleed		4 (12.5)	3 (4.8)	0.36 (0.07-1.72)	.20	0.85 (0.12-5.06)	.86
Other		1 (3.1)	2 (3.2)	1.03 (0.10-22.75)	.98	2.08 (0.12-54.38)	.60
One of the reasons for performing DRE	94						
Black stool/suspected melena		17 (53.1)	36 (58.1)	1.22 (0.52-2.89)	.65	0.66 (0.23-1.79)	.42
Red blood in stool		5 (15.6)	21 (33.9)	2.77 (0.99-9.08)	.07	2.47 (0.75-9.01)	.15
Suspected upper GI tract bleed		10 (31.2)	13 (21.0)	0.58 (0.22-1.56)	.28	0.78 (0.26-2.39)	.65
Dropping hemoglobin		8 (25.0)	27 (43.5)	2.31 (0.93-6.24)	.08	3.90 (1.34-12.81)	.02
History of GI tract bleed		4 (12.5)	10 (16.1)	1.35 (0.41-5.26)	.64	1.08 (0.28-4.75)	.92
Predominant stool color (reference: brown)	91	21 (65.6)	21 (33.9)	0.27 (0.11-0.65)	.004	0.56 (0.20-1.59)	.27
Stool consistency	94			Overall test of difference: P<.001		Overall test of difference: P<.001	
Tarry/thick		2 (6.2)	21 (33.9)	1.00 (reference)	NA	1.00 (reference)	NA
Liquid		10 (31.2)	28 (45.2)	0.27 (0.04-1.15)	.11	0.33 (0.05-1.49)	.19
Firm		20 (62.5)	13 (21.0)	0.06 (0.01-0.26)	< .001>	0.09 (0.01-0.42)	.006
Hemorrhoid present on DRE	94	2 (6.2)	7 (11.3)	1.91 (0.43-13.36)	.44	3.33 (0.65-25.81)	.18

Of the 27 patients with red blood in their stool as the indication for performing the gFOBT and who had a positive result, nine (33%) were discharged, whereas six of nine (67%) who had a negative gFOBT result were discharged (P=.14). In patients with red blood in their stool and a positive gFOBT result, the median (range) hematocrit level was 31.6% (19.9%-44.4%) for those who were admitted to the hospital or underwent observation and 42.2% (32.0%-47.7%) for those discharged (P<.001). In patients with red blood in their stool and a negative gFOBT result, the median (range) hematocrit level was 26.9% (23.8%-43.4%) for those who were admitted to the hospital or underwent observation and 39.9% (38.2%-43.4%) for those discharged (P=.44).

## Discussion

Our study evaluated ED clinicians' ability to predict the gFOBT results and whether the clinician felt that the results would change patient disposition. Our findings show that ED clinicians at our hospital are not able to consistently predict the gFOBT results. Indeed, when the ED clinicians predicted that gFOBT positivity was certain or very probable, only 79% of the corresponding gFOBTs were positive, which suggests that the gFOBT could provide some value in the comprehensive examination of patients with suspected GI tract bleeding in the ED.

The most common reason for performing the gFOBT in our study was black stool or suspected melena, followed by decreased hemoglobin level, red blood in the stool, and suspected upper GI tract bleeding, and these indications have been reported previously [10]. Previous research found the sensitivity and specificity of the gFOBT for detecting occult GI tract bleeding related to colorectal cancer to be 23.8-59.0% and 97.7-98.0%, respectively [13,14]. Notably, in our study, screening for colorectal cancer was never selected as a reason that the gFOBT was performed in the ED.

Cleveland et al. reported that removing the gFOBT from the ED was associated with a reduced number of DREs performed, which may delay diagnoses of GI tract disease or result in misdiagnoses [15]. The patients in our study did not have drug or dietary restrictions before undergoing the gFOBT in the ED, which could have contributed to false-positive and false-negative results and may have precluded the ability of ED clinicians to predict gFOBT results accurately. Thus, the results of the gFOBT should be interpreted in a broader clinical context.

Although clinicians reported that the gFOBT results would change patient disposition for most ED visits, how much the results of a single gFOBT contributed to a change in patient disposition is unknown. The gFOBT was used with other clinical assessments to determine the most appropriate patient disposition. Additionally, whether the patient dispositions were the correct endpoints of the ED visits could not be determined because we did not assess patient outcomes. Nevertheless, most ED clinicians responded that the gFOBT results would change patient disposition before performing the gFOBT, which decreased after it was performed.

Limitations

Our study has several limitations, which include selection bias. Not all ED clinicians completed a survey for each gFOBT performed, and we could not determine how many gFOBTs were performed during the study period that were not included in our analysis. Clinicians also self-reported the results of the gFOBT with no external validation due to the nature of the clinical environment. Additionally, our study did not compare the utility of the gFOBT with a control comparator. We did not account for patient diet or medications, which could affect the accuracy of the gFOBT. Because our study was performed at a single center, our results may not be generalizable to other patient populations. The small sample size resulted in low power for detecting differences in our cohort. Thus, the possibility of type II errors (ie, false-negative findings) should be considered, and we cannot conclude that no actual difference exists simply due to the occurrence of a nonsignificant P value in our small study. We also did not make any adjustment for multiple testing despite the relatively large number of statistical tests performed. Therefore, our findings must be validated in future studies.

## Conclusions

The ED clinicians at our hospital predicted positive and negative gFOBT results with an area under the receiver operating characteristic curve of 0.75 (95% CI, 0.66-0.85). When ED clinicians predicted that gFOBT positivity was certain or very probable, only 79% of the corresponding gFOBTs were positive. Our unadjusted and adjusted analyses showed that black stool or suspected melena was the most common reason ED clinicians reported that the gFOBT results would change patient disposition, and this indication may be the most important reason for performing the gFOBT in the ED setting. Overall, the general consensus among the ED clinicians at our institution is that gFOBT results will change patient disposition. Therefore, the gFOBT may play a role for a subset of ED patients, particularly those with black stool/suspected melena or red blood in the stool. However, additional studies including patient outcomes are needed to fully assess the utility of the gFOBT in the ED.

## Appendices

Emergency department clinician survey form

Place patient sticker here

-OR-

Patient name:

MRN:

DOS:

Pre-hemoccult

Number of stools in the last 24 hours:

0                      1                      2                                  ≥ 3                   unknown     

Last stool was:                 

All liquid            Somewhat formed         Formed             Unknown

Has patient taken bismuth subsalicylate (pepto bismol) in the last 48 hours?

Yes                  No                    Unsure

How many bouts of emesis in the last 24 hours?

0                      1                      2                      ≥3                    Unknown

Does patient report coffee ground or bloody emesis?

Yes                  No                                Unsure

Why are you doing a rectal exam (rank only the number of reasons you are doing the test, 1 = most important, 2= second most important, …etc.):

______     Black or suspected melena stools

______     Red blood in stools

______     Unexplained weight loss

______     Screening for colon cancer

______     Dropping hemoglobin

______     Unstable vitals

______     Suspected upper GI bleed

______     History of GI bleed in past

______     Low platelets

______     Differentiate bleeding source from Gyn or GU bleeding

______     Elevated INR (or anticoagulant use) and possible GI bleed

______     Evaluation of rectal mass

______     Evaluation of hemorrhoid

______     Other

7.     Do you think the result of the hemoccult will change the disposition of the patient?

Yes                              No

What time was the rectal exam performed?

After you perform the rectal exam and before it is hemocculted, how likely do you think the hemoccult will be positive:

A.     No chance

B.     Slight possibility

C.    Fair possibility

D.    Very probable

E.     Certain

(complete post-hemoccult questions on reverse side)

Post-hemoccult

Was the hemoccult:

A.     Negative

B.     Weakly positive

C.    Moderately positive

D.    Strongly positive

Predominant stool color:

A.     Brown

B.      Black

C.     Red/pink

D.     Yellow

E.      Orange

F.      Other

How much stool did you get to hemoccult:

A.     None

B.     Very little

C.     Sufficient

Stool consistency:

A.     Tarry/thick

B.      Liquid

C.     Firm

Did you see a hemorrhoid?

A.    Yes

B.    No

C.    Unsure

Did you see a rectal fissure?

A.    Yes

B.    No

C.    Unsure

Did the result of the hemoccult change your plan for disposition?

A.     Yes

B.     No

Comments:

## References

[1] Rui P, Kang K (2017). National Hospital Ambulatory Medical Care Survey: 2017 Emergency Department Summary Tables.

[2] Lin JS, Perdue LA, Henrikson NB, Bean SI, Blasi PR (2021). Screening for colorectal cancer: updated evidence report and systematic review for the US Preventive Services Task Force. JAMA.

[3] Sinatra MA, St John DJ, Young GP (1999). Interference of plant peroxidases with guaiac-based fecal occult blood tests is avoidable. Clin Chem.

[4] Park DI, Ryu S, Kim YH, Lee SH, Lee CK, Eun CS, Han DS (2010). Comparison of guaiac-based and quantitative immunochemical fecal occult blood testing in a population at average risk undergoing colorectal cancer screening. Am J Gastroenterol.

[5] Salisbury JD, Goodrich JG, McManus NM, Offman RP (2021). The case of the lime-green stool: a case report and review of occult blood testing in the emergency department. Clin Pract Cases Emerg Med.

[6] Sharma VK, Corder FA, Raufman JP, Sharma P, Fennerty MB, Howden CW (2000). Survey of internal medicine residents' use of the fecal occult blood test and their understanding of colorectal cancer screening and surveillance. Am J Gastroenterol.

[7] Sharma VK, Vasudeva R, Howden CW (2000). Colorectal cancer screening and surveillance practices by primary care physicians: results of a national survey. Am J Gastroenterol.

[8] Dubé C (2013). Putting an end to the misuse of the fecal occult blood test in diagnostic medicine. Can J Gastroenterol.

[9] Friedman A, Chan A, Chin LC (2010). Use and abuse of faecal occult blood tests in an acute hospital inpatient setting. Intern Med J.

[10] Ip S, Sokoro AA, Buchel A, Wirtzfeld D, Konrad G, Fatoye T, Singh H (2013). Use of fecal occult blood test in hospitalized patients: survey of physicians practicing in a large central Canadian health region and Canadian gastroenterologists. Can J Gastroenterol.

[11] von Elm E, Altman DG, Egger M (2007). The Strengthening the Reporting of Observational Studies in Epidemiology (STROBE) statement: guidelines for reporting observational studies. Ann Intern Med.

[12] Harrell FE (2001). Regression Modeling Strategies: With Applications to Linear Models Logistic Regression and Survival Analysis. https://link.springer.com/book/10.1007/978-1-4757-3462-1.

[13] Parra-Blanco A, Gimeno-García AZ, Quintero E (2010). Diagnostic accuracy of immunochemical versus guaiac faecal occult blood tests for colorectal cancer screening. J Gastroenterol.

[14] Grobbee EJ, Wisse PH, Schreuders EH (2022). Guaiac-based faecal occult blood tests versus faecal immunochemical tests for colorectal cancer screening in average-risk individuals. Cochrane Database Syst Rev.

[15] Cleveland NJ, Yaron M, Ginde AA (2010). The effect of removal of point-of-care fecal occult blood testing on performance of digital rectal examinations in the emergency department. Ann Emerg Med.

